# COVID-19 Serum Drives Spike-Mediated SARS-CoV-2 Variation

**DOI:** 10.3390/v16050763

**Published:** 2024-05-11

**Authors:** Yuanling Yu, Mengyi Zhang, Lan Huang, Yanhong Chen, Xi Wu, Tao Li, Yanbo Li, Youchun Wang, Weijin Huang

**Affiliations:** 1 Changping Laboratory, Beijing 102206, China; a0000373@cpl.ac.cn (Y.Y.); a0000277@cpl.ac.cn (L.H.);; 2 Division of HIV/AIDS and Sex-Transmitted Virus Vaccines, Institute for Biological Product Control, National Institutes for Food and Drug Control (NIFDC), Beijing 102629, China; zhangmengyi1001@163.com (M.Z.);; 3 National Institutes for Food and Drug Control, Chinese Academy of Medical Science & Peking Union Medical College, No. 9 Dongdan Santiao, Dongcheng District, Beijing 100730, China; 4 State Key Laboratory of Drug Regulatory Science, National Institutes for Food and Drug Control (NIFDC), Beijing 102629, China; 5 Beijing Yunling Biotechnology Co., Ltd., Beijing 100176, China; 6 Institute of Medical Biology, Chinese Academy of Medical Science & Peking Union Medical College, Kunming 650118, China

**Keywords:** SARS-CoV-2, COVID-19, immune evasion, infectivity

## Abstract

Neutralizing antibodies targeting the spike (S) protein of SARS-CoV-2, elicited either by natural infection or vaccination, are crucial for protection against the virus. Nonetheless, the emergence of viral escape mutants presents ongoing challenges by contributing to breakthrough infections. To define the evolution trajectory of SARS-CoV-2 within the immune population, we co-incubated replication-competent rVSV/SARS-CoV-2/GFP chimeric viruses with sera from COVID-19 convalescents. Our findings revealed that the E484D mutation contributes to increased viral resistant against both convalescent and vaccinated sera, while the L1265R/H1271Y double mutation enhanced viral infectivity in 293T-hACE2 and Vero cells. These findings suggest that under the selective pressure of polyclonal antibodies, SARS-CoV-2 has the potential to accumulate mutations that facilitate either immune evasion or greater infectivity, facilitating its adaption to neutralizing antibody responses. Although the mutations identified in this study currently exhibit low prevalence in the circulating SARS-CoV-2 populations, the continuous and meticulous surveillance of viral mutations remains crucial. Moreover, there is an urgent necessity to develop next-generation antibody therapeutics and vaccines that target diverse, less mutation-prone antigenic sites to ensure more comprehensive and durable immune protection against SARS-CoV-2.

## 1. Introduction

As of 18 February 2024, the World Health Organization has reported that the SARS-CoV-2 virus, the causative agent of the COVID-19 pandemic, has led to over 774 million cases worldwide. In response, strategies such as vaccines, convalescent plasma therapy, and monoclonal antibodies (mAbs) have been pivotal in attempting to mitigate the pandemic’s impact. These interventions primarily target the SARS-CoV-2 spike (S) protein, essential for viral attachment and entry into host cells. The S protein comprises two subunits: S1, which includes the N-terminal domain (NTD) and receptor binding domain (RBD) for angiotensin-converting enzyme 2 (ACE2) receptor recognition and binding, and S2, which is responsible for facilitating virus–host cell membrane fusion through a six-helical bundle formation via the two-heptad repeat domain [1,2,3].

A critical consideration In developing vaccines and mAb therapies is the virus’s potential for evolution under immune pressure, potentially escaping herd immunity in populations that have recovered or been vaccinated. With millions infected by SARS-CoV-2, the levels of neutralizing antibodies vary widely. Interactions between the virus and suboptimal concentrations of neutralizing antibodies may drive viral mutations, enhancing fitness or enabling immune evasion. Particularly, immune-compromised individuals treated with mAb drugs or convalescent plasma have shown multiple site mutations during prolonged infection, including H69/V70 deletions in the NTD, E484K/A, and N501Y substitutions in the RBD and a D796H mutation in the S2 subunit [4,5]. The rapid predominance of the SARS-CoV-2 variant JN.1 over its predecessor BA.2.86, attributed to an additional L455S mutation in the RBD, underscores the virus’s capacity for rapid evolution under immune pressure [6].

Replication-competent rVSV/SARS-CoV-2/GFP viruses, which mimic SARS-CoV-2 spike protein-mediated infection and are neutralized by S-specific mAbs in vivo [7], were used to assess the evolution of the spike protein in the presence of mAbs or polyclonal antibodies [8,9,10]. While previous studies predominantly focused on mutations within the RBD under antibody selective pressure and their impact on viral antigenicity [8,9,10], less attention has been paid to mutations in the S2 subunit and to changes in the infectivity of variant strains. In this study, by selecting with sera from individuals who recovered from wild-type (WT) SARS-CoV-2 infection, we identified escape mutants within the NTD, RBD, and S2 subunit, providing a comprehensive view of spike protein evolution under immune pressure. Notably, substitutions at residue E484 of the S protein conferred resistance to both convalescent and vaccinated sera, consistent with previous findings that suggest certain individuals produce neutralizing antibodies targeting this specific site on the RBD. Importantly, the L1265R/H1271Y double mutation in the S2 subunit significantly enhanced viral infectivity in 293T-hACE2 and Vero cells. These results indicate that under immune pressure, SARS-CoV-2 can accumulate mutations that facilitate immune evasion or increase infectivity. It is therefore crucial to identify mutations that significantly alter viral behavior, monitor the prevalence of these mutations, and develop vaccines and therapeutic antibodies targeting distinct, less mutation-susceptible antigenic sites to ensure long-term and robust immune protection. Additionally, our study underscores the importance of considering pre-existing immunity when developing replication-competent vaccines. Pre-existing immunity might induce mutations in the vaccine strain.

## 2. Materials and Methods

### 2.1. Cell Lines and Serum Samples

HEK293T (American Type Culture Collection [ATCC], CRL-3216), Huh-7 (Japanese Collection of Research Bioresources, Cat No. 0403), Vero (ATCC, CCL-81), and Vero E6 (ATCC, CRL-1586) cell lines were maintained in Dulbecco’s Modified Eagle Medium (DMEM) enriched with 10% fetal bovine serum (FBS) and 100 U/mL penicillin-streptomycin. HEK293T cells stably expressing the human ACE2 receptor (HEK293T-hACE2) were cultured in DMEM supplemented with 10% FBS, 100 U/mL penicillin-streptomycin, and 15 µg/mL blasticidin for selection [11]. All cell lines were incubated at 37 °C with 5% CO_2_ and passaged every 2–3 days.

### 2.2. Serum Samples

Convalescent serum samples obtained from patients who had recovered from SARS-CoV-2 (Wuhan-Hu-1) infections, were kindly supplied by Dr. Xiaowang Qu of Nanhua University (*n* = 24). The study protocol was approved by the Institutional Ethical Review Board of The Central Hospital of Shaoyang (V. 1.0, 20200301). Each participant signed a written consent form.

Serum samples from vaccinated individuals were generously provided by Dr. Jiankai Liu of Shenzhen Kangtai Biological Products Co., Shenzhen, China (*n* = 18). These samples were obtained from volunteers who had received the inactivated virus vaccine (KCONVAC, Shenzhen Kangtai Biological Products Co.; Chinese Clinical Trial Registry: ChiCTR2000038804), which was approved for emergency use in China in May 2021. The samples were collected 14 days after the completion of the standard immunization regimen.

### 2.3. Generation of Replication-Competent VSV/SARS-CoV-2/GFP Chimeric Virus

The rVSV/SARS-CoV-2/GFP chimeric virus was generated from cDNA clones following previously described methods with modifications [7]. Briefly, a plasmid bearing the T7 promoter upstream of the VSV anti-genome (Indiana serotype) was engineered by replacing the G gene with a codon-optimized SARS-CoV-2 spike gene from the Wuhan-Hu-1 isolation (GenBank: MN908947), also referred to as the wild-type (WT) strain, and by inserting an eGFP reporter gene as an independent transcription unit preceding the spike gene. The replication-competent VSV-based pseudovirus was then rescued using a plasmid-based method [7,12]. The growth kinetics of the recovered virus are detailed and can be viewed in Figure S1.

### 2.4. Selection of Viruses in the Presence of COVID-19 Serum In Vitro

To select for SARS-CoV-2 variants resistant to COVID-19 convalescent serum, 1 × 10^5^ focus-forming units per ml (FFU/mL) of the rVSV/SARS-CoV-2/GFP virus were incubated with three-fold serial dilutions of convalescent serum (ranging from 0.11× to 9× ED_50_) at 37 °C for 1 h. The virus–serum mixtures were then exposed to 2 × 10^4^ Vero cells seeded in 96-well plates. After 3 days, GFP-positive cells were counted by ELISPOT, and supernatants from these first-passage (P1) cultures were collected for further incubation and infection assays as described. The rVSV/SARS-CoV-2/GFP viruses underwent up to eight passages with serum. An increase in GFP-positive cells, as detected by ELISPOT, indicated the outgrowth of potential immune escape variants.

### 2.5. Sequence Analyses

Viral RNA was isolated from the supernatants collected at passages 2, 5, 7, and 8, utilizing the RNeasy Mini Kit (QIAGEN, Hilden, Germany). Subsequent cDNA synthesis was facilitated by the OneStep RT-PCR Kit (QIAGEN), employing primers targeting the VSV-M and VSV-L genes. The complete sequence encoding the S protein was then amplified with KOD FX Neo (Takara) and primers designed to flank the S gene. The resulting PCR products underwent deep sequencing at the HaploX Genomics Center, where mutation frequencies were also analyzed. In brief, the raw NGS data were processed using the open-source software fastp (version 0.20.0) to filter out low-quality reads. These data were then aligned to the reference sequence (S sequence in rVSV/SARS-CoV-2/GFP) using the mpileup command in Samtools (version 1.3.1) for accurate mutation detection. The mutation detection results were further refined using bcftools with criteria: “QUAL > 20 && DP > 4 && MQ > 30”. Mutation frequencies were calculated based on the variant allele frequency (VAF), which is the ratio of the allelic depth to the tatal depth. For precise identification of mutations within dominant viral variants (DVs), the PCR products were cloned into T vectors, with specific mutations identified via Sanger sequencing conducted by SinoGenoMax.

### 2.6. Neutralization Assay

Neutralization assays utilized single-cycle VSV/SARS-CoV-2/Fluc viruses, either expressing the WT SARS-CoV-2 spike protein or its mutated variants. The assay followed established methodologies [13,14]. Test serum samples were serially diluted and incubated with pseudotyped viruses at 37 °C for 1 h, followed by the addition of trypsinized Huh-7 cells. After 24 h of incubation with 5% CO_2_ at 37 °C, the relative light unit (RLU) was measured according to the manufacturer’s instructions (PerkinElmer, Waltham, MA, USA).

### 2.7. Infectivity Assay

The infectivity of single-cycle VSV/SARS-CoV-2/Fluc viruses, expressing either the WT SARS-CoV-2 spike protein or its mutated variants, was assessed as described in our earlier work [15]. Following RT-PCR quantification, viruses were diluted to equal particle counts and added to 96-well plates containing freshly trypsinized Vero or HEK293T-hACE2 cells. After 24 h of incubation with 5% CO_2_ at 37 °C, the RLUs were measured following the PerkinElmer manual. The relative infectivity of each variant compared to the WT strain was calculated as RLU (variant)/RLU (WT).

## 3. Results and Discussion

**Figure 1:**
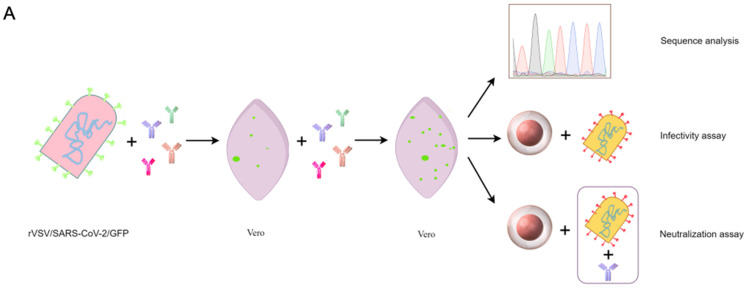

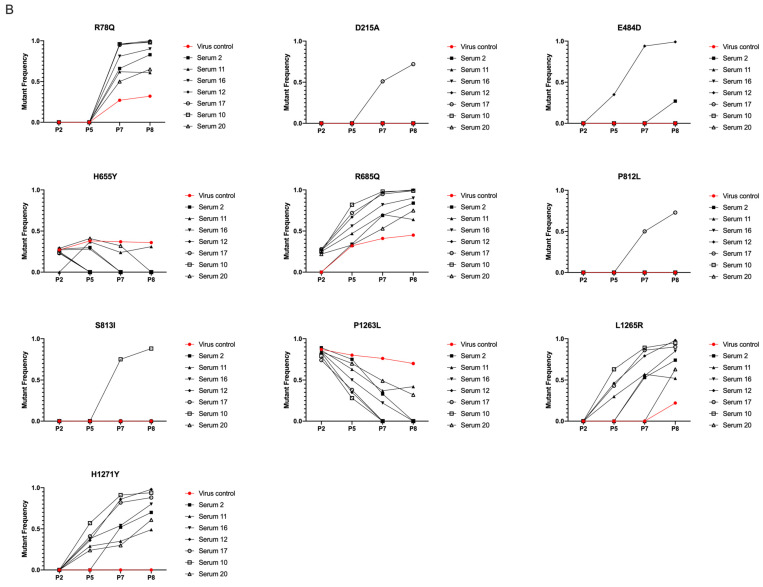
Evolution of the SARS-CoV-2 spike (S) protein under immune pressure from convalescent serum. (**A**) Diagram illustrating the selection process for escape variants and the characterization of their mutations. (**B**) The analysis of sequences encoding the S protein during passage of rVSV/SARS-CoV-2/GFP in Vero cells with convalescent serum. The mutation frequency was analyzed following the second (p2), fifth (p5), seventh (p7), and eighth (p8) passages.

Preventive vaccines and therapeutic antibodies targeting the SARS-CoV-2 spike protein have emerged as effective strategies against the COVID-19 pandemic. However, the efficacy of these measures is challenged by viral mutations that may confer antibody resistance. Here, we utilized rVSV/SARS-CoV-2/GFP chimeric viruses to investigate the evolution of SARS-CoV-2 under immune pressure exerted by COVID-19 convalescent serum (Figure 1A). An increase in GFP-positive cells detected by ELISPOT was observed, indicative of potential immune escape variant outgrowth, particularly when exposed to serum samples 2, 12, 17, and 10 in passages 7 or 8 (Figure S2). In contrast, serum 20 did not induce escape variants in passage 8, hinting at a broader neutralization spectrum that does not rely on one dominant antibody specificity. The lack of escape variants with serum 11 and 16 might simply be due to the lower potency of these serum samples (Figure S2, Table S1).

**Figure 2:**
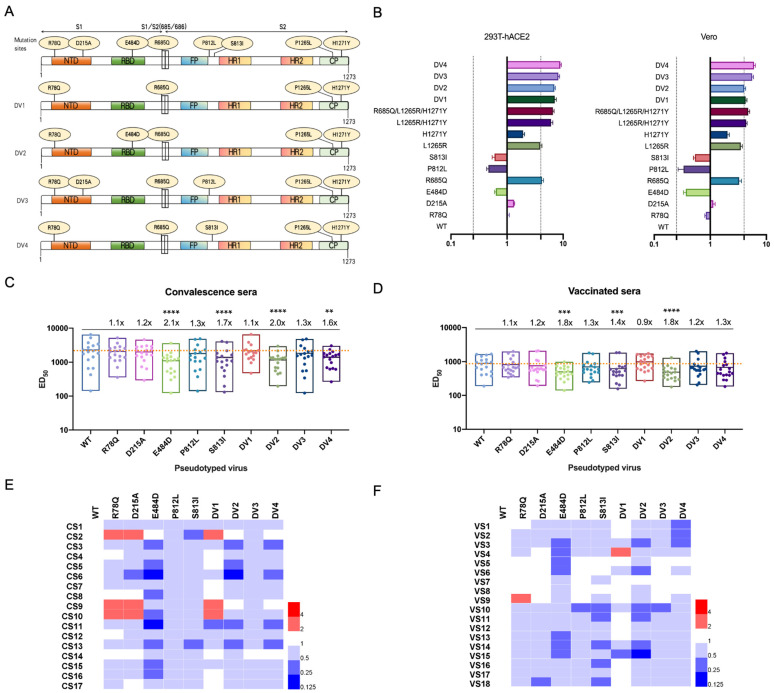
Characterization of mutation identified under immune pressure from convalescent serum. (**A**) Illustration of spike protein mutation in immune escape variants. (**B**) Relative infectivity of identified mutations compared to the reference wild-type (WT) strain. The efficiencies of infecting 293T-hACE2 or Vero cells were assessed using single-cycle VSV/SARS-CoV-2/Fluc viruses. Error bars represent the mean ± SEM of three replicates. The dashed lines indicate the threshold value of a 4-fold difference in infectivity. (**C**,**D**) Antigenic analysis of the mutations with a panel of convalescent serum (*n* = 17) (**C**) and vaccinated serum (*n* = 18) (**D**). The data represent results from three replicates. Fold changes relative to the WT, and statistical significance are annotated above each group. The dashed line represents the mean serum response to the WT strain. One-way ANOVA and Holm–Sidak’s multiple comparison tests were used to analyze the differences between groups. (**E**,**F**) Using Heatmap Illustrator (HemI), we generated a heatmap based on the fold changes in ED_50_ values of mutant strains compared to the WT. The red and blue boxes indicate the increase or decrease in the neutralization activity as shown in the scale bar. The top of the heatmap labels the specific mutations, while the left side identifies the corresponding serum samples. A *p*-value of <0.05 was considered to be significant. ** *p* < 0.01, *** *p* < 0.001, **** *p* < 0.0001. DV Dominant viral variant, ED_50_ 50% effective dilution, CS convalescence serum, VS vaccinated serum.

The sequence analysis indicated that variants R78Q, R685Q, L1265R, and H1271Y became increasingly prevalent following serum exposure, whereas variants H655Y and P1263L exhibited a decline (Figure 1B). Furthermore, the mutation frequencies of H655Y and P1263L in the virus control were slightly higher than those observed in the presence of serum, suggesting that genetic drift, rather than natural selection, may be driving the evolution of these sites. Notably, exposure to serum 17 facilitated the enrichment of variants D215A and P812L in passage 7, reaching frequencies of 0.72 and 0.73, respectively, in passage 8. The E484D variant was observed in passage 5 in the presence of serum 12 and in passage 8 with serum 2, while the S813I variant was enriched in passage 7 with serum 10 (Figure 1B). The recurrent involvement of the E484 epitope underscores its significance as a critical antigenic site within the S protein. Two serum samples induced the E484D mutation, suggesting that the enrichment of this variant may be a consequence of natural selection. For other sites, such as D215A, P812L, and S813I, additional replicates using the same specific serum sample would provide more definitive evidence regarding the evolutionary mechanisms driving these mutations. Through Sanger sequencing, dominant viral variants (DVs) were identified in the presence of serum: DV1, containing mutations R78Q, R685Q, L1265R, and H1271Y; DV2, which includes E484D in addition to DV1; DV3, which emerged in response to serum 17 and incorporated D215A and P812L alongside DV1; and DV4, detected with serum 10, featuring a total of five mutations, including S813I (Figure 2A, Table S1). These findings underscore the varied immune-driven mutational landscapes engendered by distinct convalescent serum exposures. The differential responses of SARS-CoV-2 to population-specific immune backgrounds highlight the virus’s adaptive potential under varying immune pressures, suggesting unique evolutionary paths for viral adaptation.

To assess the impacts of identified variants on viral functionality, we generated single-cycle VSV/SARS-CoV-2/Fluc pseudoviruses, each harboring specific mutations. Infectivity assays were conducted using 293T-hACE2 and Vero cells, both of which are susceptible to SARS-CoV-2. The assays revealed that individual mutations did not significantly alter viral infectivity. Conversely, combinations of mutations, particularly those observed in dominant viral variants, substantially increased infectivity, highlighting the L1265R and H1271Y mutations in the S protein’s cytoplasmic tail as pivotal enhancers of viral function (Figure 2B). This corroborates prior studies suggesting that alterations in the cytoplasmic tail, such as truncations, can significantly increase infection efficiency relative to the full-length S protein [3,16,17]. Of note, the infectivity measured here is obtained through VSV-based pseudovirus assays. The changes in the cytoplasmic tail may only be critical in the VSV system and may not provide any advantages to the authentic virus. Further investigation is necessary to elucidate the molecular mechanisms driving this increased infectivity. Notably, the R685Q mutation, located within the polybasic furin-like cleavage site, was exclusively found in convalescent serum and during virus passages, pointing to its possible role in cell culture adaptation. This pattern mirrors observations in cell culture passages of VSV-based pseudotyped and SARS-CoV-2 viruses, underscoring the potential for such a mutation to reflect adaptations to cell culture conditions [9,18].

Further investigations into the mutations’ effects on viral antigenicity through neutralization assays with a panel of convalescent and vaccinated sera are needed. Variants R78Q and D215A, located within the NTD, did not significantly impact the neutralization efficacy of polyclonal antibodies. In contrast, the E484D mutation within the RBD resulted in the most notable decrease in neutralization, exhibiting a 2.1-fold and 1.8-fold decrease in efficacy compared to the WT strain, respectively (Figure 2C,D). The neutralization sensitivity of the E484D variant was reduced in 9 of 17 convalescent sera and 7 of 18 vaccinated sera, with a decrease in neutralization potency by more than two-fold (Figure 2E,F). This highlights the potential for individual escape mutants to resist neutralization, suggesting the antigenic site repertoire of the RBD is limited in some individuals. Notably, substitutions P812L and S813I within the amino acid region 810–816 (SKPSKRS), a predicted dominant B cell epitope with cross-reactivity to SARS-CoV [19,20], resulted in a 1.3–1.7 fold decrease in the neutralization efficacy of sera samples (Figure 2C,D). Neutralization assays with pseudotyped viruses carrying dominant strain mutations revealed that variant DV1 did not significantly impact the neutralizing activity of polyclonal antibodies, aligning with evolutionary outcomes under polyclonal antibody pressure and suggesting a limited enrichment of immune escape variants. However, the incorporation of E484D (DV2) exhibited resistance to both convalescent and vaccinated sera, leading to a 2.0- and 1.8-fold reduction in neutralization, respectively. Conversely, the introduction of D215A and P812L mutations (DV3) did not significantly change the neutralization activity of sera samples. Nevertheless, introducing the S813I mutation to DV1 (forming DV4) subtly reduced neutralization susceptibility by 1.3- to 1.6-fold compared to the WT strain (Figure 2C,D). Interestingly, both the Beta variant and the recently emerged BA.2.87.1 variant carry the D215 mutation, with the latter’s resistance to humoral immunity attributed to two deletions (Δ15–26 and Δ136–146) in the NTD [21,22]. These observations highlight the necessity of monitoring mutations within the NTD as well as the RBD. Remarkably, the E484 residue has exhibited a range of mutations under the pressure of convalescent sera or mAbs [8,9,10,23], and this variability may be due to E484 being targeted by antibodies derived from IGHV3-53 and IGHV3-66 germlines, which are common in antibodies directed against the RBD [24]. The diversity of amino acid changes at this position, each with a unique antibody binding mode, indicates a degree of homogeneity among neutralizing antibodies generated across different individuals. Collectively, these results underline the critical need for a comprehensive survey of SARS-CoV-2 neutralizing specificities post-natural infection or vaccination. It also advocates for the development of vaccines and therapeutic antibodies targeting a broader range of antigenic sites, particularly those less susceptible to mutation, to ensure lasting and effective immune protection.

The variants identified were generated during the replication of rVSV/SARS-CoV-2/GFP under the selective pressure of polyclonal antibodies in vitro. As the vaccinated and infected population grows, monitoring the prevalence of these immune pressure-selected mutations within naturally circulating SARS-CoV-2 becomes crucial. Utilizing the GISAID SARS-CoV-2 databases, we analyzed the frequency of these selected mutations in natural viral populations up to March 6, 2024. It was observed that the SARS-CoV-2 S variants identified in this study are currently found at low frequencies within the circulating virus populations. Specifically, only five variants containing the R685Q mutation were detected, and the mutation frequencies for the other seven mutation sites in natural populations were low. Notably, the P812L mutation in the S2 subunit exhibited the highest natural mutation frequency at 0.075%, followed by S813I in the S2 subunit and D215A in the NTD region, with mutation frequencies of 0.031% and 0.025%, respectively (Table 1). These findings suggest that the neutralizing activities of the serum samples evaluated have not driven a strong selection pressure on the naturally circulating sequences of SARS-CoV-2 thus far. While natural selection may favor more advantageous mutations, the low frequencies of the mutations observed in this study suggest that genetic drift could also significantly influence their prevalence in natural populations. This dual perspective enriches our understanding of the complex dynamics governing viral evolution under immune pressure and environmental changes. Despite the low frequency of these mutations in circulating populations, the vigilant monitoring of immune escape mutation sites is warranted. This is because mutations not currently prevalent have the potential to become widely spread upon acquiring certain additional mutations. For instance, the BA.2.86 variant was initially limited in its spread but became predominant after acquiring the L455S mutation, evolving into the JN.1 strain. This underscores the importance of ongoing surveillance and research to understand the potential impact of emerging mutations on the trajectory of the COVID-19 pandemic.

## 4. Conclusions

This study elucidates the adaptive evolution of SARS-CoV-2 under immune pressure, highlighting how neutralizing antibodies from natural infection can drive the enrichment of escape variants. Specifically, we identified key mutations, such as E484D and the L1265R/H1271Y double mutation, that demonstrate the virus’s capacity to evade immune responses and enhance infectivity. Although these mutations are not widely prevalent in circulating variants, their existence stresses the imperative to continuously track resistance mutations, akin to monitoring for antiviral and antibiotic resistance in other pathogens. Furthermore, this study emphasizes the importance of incorporating evolutionary considerations into the design of future public health measures against COVID-19. It also underscores the urgent need for next-generation vaccines and antibody therapeutics targeting broader and less mutation-prone antigenic sites, thereby ensuring robust and enduring protection against the evolving threat of SARS-CoV-2.

## Figures and Tables

**Table 1:** Frequency distribution of selected mutations in natural populations.

Mutation	Number of Viruses	Mutation Frequency
R78Q	114	0.001%
D215A	4077	0.025%
E484D	1711	0.010%
R685Q	5	0.000%
P812L	12468	0.075%
S813I	5095	0.031%
L1265R	156	0.001%
H1271Y	164	0.001%

## Data Availability

The data presented in this study are available on request from the corresponding author. The data are not publicly available due to privacy.

## Supplementary Materials

The following supporting information can be downloaded at: https://www.mdpi.com/article/10.3390/v16050763/s1, Figure S1: Growth kinetics of the rVSV/SARS-CoV-2/GFP viruses in Vero cells at a multiplicity of infection (MOI) of 0.1; Figure S2: Selection of SARS-CoV-2 spike (S) protein escape mutants; Table S1: Overview of characteristics in COVID-19 convalescent sera and dominant variants identified within convalescent serum samples.
