# Supplementary material for: COVID-19 Serum Drives Spike-Mediated SARS-CoV-2 Variation
Source: Viruses. 2024 May 11;16(5):763. doi: 10.3390/v16050763 (PMC11126028; doi:10.3390/v16050763)
Supplement: Supplementary file 1 [file viruses-16-00763-s001.zip › viruses-2964699-supplementary.pdf]

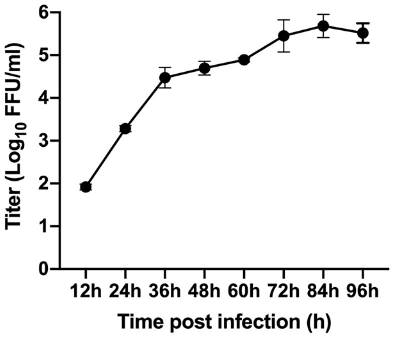

Figure S1. Growth kinetics of the rVSV/SARS-CoV-2/GFP viruses in Vero cells at a multiplicity of infection (MOI) of 0.1. Viral titers were quantified using a focus-forming assay and are expressed as focus-forming units per milliliter (FFU/ml). Error bars represent the standard deviation of the mean (n=3).

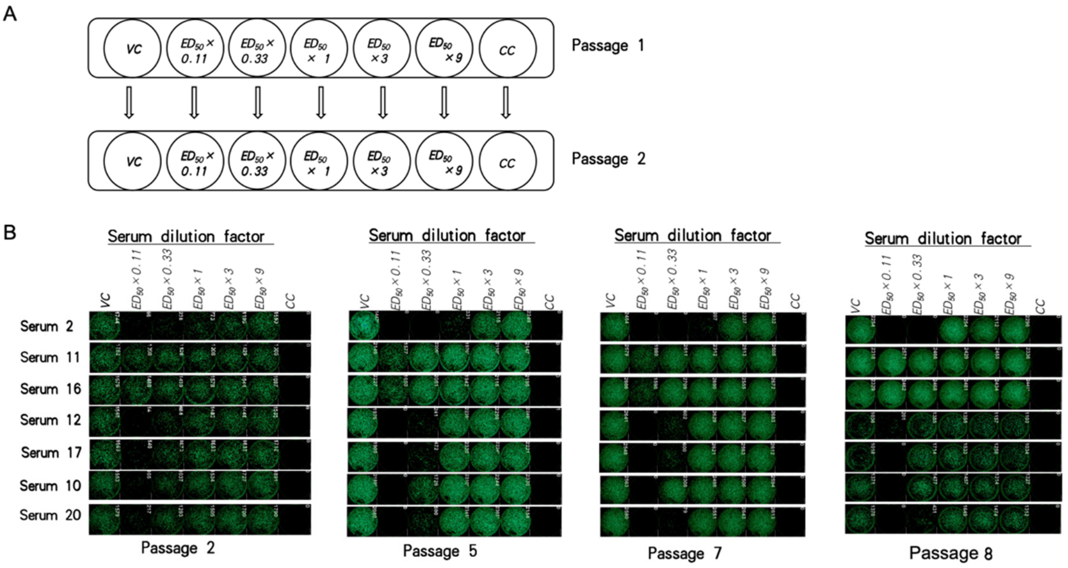

Figure S2. Selection of SARS-CoV-2 spike (S) protein escape mutants. (**A**) Schematic representation of the 96-well plate format used for selecting SARS-CoV-2 escape mutants with convalescent serum obtained from patients who had recovered from SARS-CoV-2 (Wuhan-Hu-1) infections. (**B**) Quantification of GFP-positive cells at various passages and serum dilution factors using ELISPOT. VC virus control, CC cell control, ED_{50} 50% effective dilution.

Table S1. Overview of characteristics in COVID-19 convalescent sera and dominant viral variants identified within convalescent serum samples.

Serum ID	Neutralization ED_{50}	Binding titer RBD	Binding titer S2	Dominant viral variant (DV)
2	69	160	640	DV2 (R78Q/E484D/R685Q/L1265R/H1271Y)
11	38	40	320	DV1 (R78Q/R685Q/L1265R/H1271Y)
16	43	160	1280	DV1 (R78Q/R685Q/L1265R/H1271Y)
12	322	1280	1280	DV2 (R78Q/E484D/R685Q/L1265R/H1271Y)
17	286	640	320	DV3 (R78Q/D215A/R685Q/P812L/L1265R/H1271Y)
10	435	640	640	DV4 (R78Q/R685Q/S813I/L1265R/H1271Y)
20	492	≥2560	640	DV1 (R78Q/R685Q/L1265R/H1271Y)

ED_{50} 50% effective dilution, RBD receptor binding domain, S spike.
